# Staphylococcal Superantigens Stimulate Immortalized Human Adipocytes to Produce Chemokines

**DOI:** 10.1371/journal.pone.0077988

**Published:** 2013-10-30

**Authors:** Bao G. Vu, Francoise A. Gourronc, David A. Bernlohr, Patrick M. Schlievert, Aloysius J. Klingelhutz

**Affiliations:** 1 Department of Microbiology, University of Iowa^,^ Carver College of Medicine, Iowa City, Iowa, United States of America; 2 Department of Biochemistry and Molecular Biology/Biophysics, University of Minnesota, Medical School, Minneapolis, Minnesota, United States of America; University of Edinburgh, United Kingdom

## Abstract

**Background:**

Human adipocytes may have significant functions in wound healing and the development of diabetes through production of pro-inflammatory cytokines after stimulation by gram-negative bacterial endotoxin. Diabetic foot ulcers are most often associated with staphylococcal infections. Adipocyte responses in the area of the wound may play a role in persistence and pathology. We studied the effect of staphylococcal superantigens (SAgs) on immortalized human adipocytes, alone and in the presence of bacterial endotoxin or staphylococcal α-toxin.

**Methodology/Principal Findings:**

Primary non-diabetic and diabetic human preadipocytes were immortalized by the reverse transcriptase component of telomerase (TERT) and the E6/E7 genes of human papillomavirus. The immortal cells were demonstrated to have properties of non-immortalized pre-adipocytes and could be differentiated into mature and functional adipocytes. Differentiated adipocytes exposed to staphylococcal SAgs produced robust levels of cytokines IL-6 and IL-8, but there were no significant differences in levels between the non-diabetic and diabetic cells. Cytokine production was increased by co-incubation of adipocytes with SAgs and endotoxin together. In contrast, α-toxin alone was cytotoxic at high concentrations, but, at sub-cytotoxic doses, did not stimulate production of IL-6 and IL-8.

**Conclusions/Significance:**

Endotoxin has been proposed to contribute to diabetes through enhanced insulin resistance after chronic exposure and stimulation of adipocytes to produce cytokines. Our data indicate staphylococcal SAgs TSST-1 and SEB alone and in combination with bacterial endotoxin also stimulate adipocytes to produce cytokines and thus may contribute to the inflammatory response found in chronic diabetic ulcers and in the systemic inflammation that is associated with the development and persistence of diabetes. The immortal human pre-adipocytes reported here will be useful for studies to understand further the mechanism by which toxins are involved in wound healing and the development and clinical manifestations of obesity and diabetes.

## Introduction

Adipocytes are emerging as important participants in the development of diabetes mellitus type II (DMII) [1]. Secretion of free-fatty acids and hormones by adipocytes play a central role in metabolism, gluconeogenesis, and inflammation. During the expansion of adipose tissue, such as occurs in obesity, persistent inflammatory responses may occur accompanied by an upregulation of pro-inflammatory cytokines released by adipocytes. This may lead to chronic subclinical inflammation as well as insulin resistance; and ultimately contribute to the development of DMII. Additionally, high fat diets may result in chronic increases of systemic endotoxin (lipopolysaccharide, LPS) levels derived from leakage across the gastrointestinal tract from normal microbiota [2], [3]. Endotoxin directly induces pro-inflammatory cytokine production (for example IL-6, IL-8, and TNF-α) in adipocytes via toll-like receptor 4 (TLR-4) activation, with resultant glycemia and insulinemia in experimental animals [4], [5]. Similar correlative findings have been observed in DMII patients where higher levels of endotoxin were found in serum and more activated pro-inflammatory signaling pathways were found in adipose tissue as compared to serum and tissue from non-diabetic individuals [6].

While DM can have multiple complicating sequelae, infections in diabetic foot ulcers (DFUs) are among the most frequent and serious complications [7]. From 15–25% of DM patients will develop DFUs in their lifetimes, and among them, 1 in 6 will succumb within a year after onset of infection [8]. Although DFU infections are often polymicrobial, the gram-positive bacterium *Staphylococcus aureus* remains the most common isolate. For most of these *S. aureus* infections, the DM patient is likely to be the source of the infection, with organisms originating on mucosal surfaces, such as nares, vagina, and gastrointestinal tract, and from skin surfaces. This means many DM patients are colonized with *S. aureus* prior to DFU development.

*S. aureus* produces many virulence factors that have cytokine-stimulating properties in common with endotoxin [9]. Among these, superantigen (SAg) exotoxins have been shown to have significant roles in contributing to major illnesses caused by the organism [9], [10], [11]. The hallmark activities of SAgs are their capacities to induce fever and hypotension, like endotoxin, but through a different and unique mechanism of T cell and macrophage activation [9], [12]. SAgs cross-bridge variable regions of the β-chain of T cell receptors (Vβ-TCRs) and α- and/or β-chains of major histocompatibility complex (MHC) class II molecules, leading to massive cytokine production by both T cells and macrophages [9], [10].

SAgs also interact with epithelial cells to promote inflammatory responses [9], [13]. For example, the SAg toxic shock syndrome toxin-1 (TSST-1) directly induces IL-6, IL-8, MIP-3α, and TNF-α production in human vaginal epithelial cells [13]. It is thus possible that SAgs, like endotoxin, could stimulate adipocytes to produce cytokines, which may also contribute to development of DMII. Emerging evidence indicates that intradermal adipocytes are critical for proper skin wound healing [14]. Because of their pro-inflammatory activities, it is also hypothesized that SAgs in chronic DFU infections contribute to the maintenance of DFUs by adipocyte-induced inflammation and consequent inhibition of wound healing.

The present study was undertaken to investigate the effects of *S. aureus* SAgs and α-toxin on human adipocytes. Many studies show that SAgs synergize with endotoxin in causing massive cytokine production, and this process may contribute to the development of the cytokine storm in TSS [9], [15], [16]. Similar responses may occur in DMII patients and patients with DFUs. Thus, this study also examines the effect of SAgs in combination with endotoxin on adipocyte production of pro-inflammatory cytokines. Of potential great value, we developed high-quality immortalized human pre-adipocytes that can easily be matured into adipocytes. These cells may provide a useful model system for studying the effects of potential toxic agents on adipocyte-associated inflammatory responses.

## Materials and Methods

### Development of DPAD and NPAD Cell Lines

Primary pre-adipocytes from a single diabetic (D) and a single non-diabetic (N) donor were purchased from Lonza, Portsmouth, NH. The cells were thawed from −80°C, distributed into three 60 mm tissue culture plates (BD-Falcon, Franklin Lakes, NJ), and cultured in pre-adipocyte media (Lonza) prepared according to the manufacturer’s instructions. Cells on one of the plates were transduced with a combination of TERT-BABE-Hygro [17] and 16E6/E7-LXSN [18] retroviruses overnight in 8 ug/ml polybrene [19]. Another plate was transduced with vectors only (BABE-Hygro and 16E6/E7-LXSN), and the third plate was mock-transduced. On the following day, the media on the cells was changed and, on the next day, the cells were diluted 1/3, followed by selection on the following day with 20 ug/ml hygromycin for 8 days. Cells that were alive after this selection were split 1∶2 and then selected with 300 ug/ml G418. Only those cells that had been transduced with TERT and E6/E7 survived and proliferated. These were designated DPADs (diabetic pre-adipocytes) and NPADs (non-diabetic pre-adipocytes). Proliferating cells were tested to ensure they were *Mycoplasma*-negative and cryopreserved at early passage. Cells were passaged at 1∶4 splits every 4 days in Lonza pre-adipocyte media.

### Adipocyte Differentiation

Cells were grown to confluency in pre-adipocyte media in 6-well or 96-well plates and then changed to differentiation media (Lonza) prepared according to the manufacturer’s instructions. The cells were maintained in this medium for 10 days to allow differentiation to adipocytes. The differentiated cells will be referred to as DDADs (diabetic differentiated adipocytes) and NDADs (non-diabetic differentiated adipocytes).

### Photomicrographs

Cells were stained with AdipoRed (Lonza) according to the manufacturer’s instructions. Photomicrographs were taken using a Nikon Eclipse TE200 microscope at 200X and processed by Image J software.

### Assessment of Adipocyte Differentiation Markers by Quantitative RT-PCR

RNA was collected from exponentially growing NPAD and DPAD, and from differentiated NDAD and DDAD cultures. For RNA collection, the plates were washed with phosphate-buffered saline (PBS; 0.005 NaPO_4_, 0.15 M NaCl), and total RNA was extracted from cells using 1 ml of Trizol Reagent (Invitrogen, Grand Island, NY) according to the manufacturer’s instructions, followed by purification with RNeasy Mini Columns (Qiagen, Redwood City, CA). RNA was reverse-transcribed using a Retroscript kit (Ambion, Foster City, CA) with random decamers as recommended by the manufacturer. Quantitative reverse transcriptase PCR (q-RT-PCR) was performed as described [20], [21]. q-RT-PCR was performed in triplicate using the ABI PRISM 7700 Sequence Detection System with standard cycling in SYBR-Green PCR Master Mix (Applied Biosystems, Foster City, CA). The assayed genes and the primers that were used for PCR are listed in Table 1. Expression levels were normalized to glyceraldehyde-3-phosphate-dehydrogenase (GAPDH) and relative to exponentially growing NPADs.

**Table 1:** Genes that were analyzed by q-RT-PCR with respective primers.

Gene Name	Forward Primer (5′-3′)	Reverse Primer (5′-3′)
**CEBPA** (CCAAT/enhancer binding protein, alpha)	ACT AGG AGA TTC CGG TGC CTC	GAA TTC TCC CCT CCT CGC AG
**PPARG** (peroxisome proliferator-activated receptor gamma)	GCC CAG GTT TGC TGA ATG TG	TGA GGA CTC AGG GTG GTT CAG
**LPL** (lipoprotein lipase)	CGA GCG CTC CAT TCA TCT CTT C	CCA GAT TGT TGC AGC GGT TC
**LEP** (leptin)	CTT CAC GTG CTG GCC TTC TC	ACC TCT GTG GAG TAG CCT GAA G
**ADIPOQ** (adiponectin)	GCT CAG CAT TCA GTG TGG GA	GTA CAG CCC AGG AAT GTT GC
**CFD** (complement factor D; adipsin)	GCT GGG GCA TAG TCA ACC AC	ATT GCT CTC CGC GCA CAT
**FABP4** (fatty-acid-binding-protein-4;ap2)	AAC TGG TGG TGG AAT GCG TC	TGC GAA CTT CAG TCC AGG TC
**CCL2** (chemokine C-C motif ligand 2;MCP- 1)	GAG GCT CGC GAG CTA TAG AAG	GGT TTG CTT GTC CAG GTG GTC
**GAPDH** (glyceraldehyde-3-phosphate dehydrogenase)	AAG GTC ATC CAT GAC AAC TTT G	GTA GAG GCA GGG ATC ATC TTC T

### Toxin Purification

The SAg purification method is detailed elsewhere [22]. In short, *S. aureus* RN4220::PCE107 (TSST-1) and MNHoch (SEB) were cultured in beef heart (BH) media (with high aeration) for 48 h. Cultures were then precipitated in absolute ethanol (80% final volume) and SAgs purified to homogeneity by thin-layer isoelectric focusing.

The same method was used for α-toxin purification except, *S. aureus* MNPE strain was grown in BH media (with high aeration) for 24 h [23]. The culture was then treated ammonium sulfate to 80% saturation to precipitate α-toxin, which was resolubilized in distilled water and purified to homogeneity by thin layer isoelectric focusing.

Sodium dodecyl sulfate-polyacrylamide gel electrophoresis (SDS-PAGE) [24] and Western immunoblot [25] with specific antibodies raised against each toxin were used to evaluate the purified toxins.

### Endotoxin Purification

Endotoxin was purified according to the hot phenol extraction method from *Salmonella* Typhimurium [26]. The endotoxin preparation was demonstrated to be biologically active with use of the *Limulus* amoebocyte assay and pyrogen testing (minimum pyrogenic dose was 0.01 µg/ml).

### ELISAs for Chemokines and Cytokines

DDADs and NDADs were treated with SAgs (50 and 100 ug/ml), α-toxin (0.0005–10 ug/ml), or endotoxin (2.5–10 ng/ml) for 24 h at 37°C in 7% CO_2_. The culture supernates were collected for IL-6 and IL-8 quantification by ELISA (Quantikine® kits, R and D Systems, Minneapolis, MN).

### Cytotoxicity Assay

After treatment with various concentrations (0.1–10 ug/ml) of α-toxin for 24 h, cells were washed and resuspended in PBS. CellTiter 96® Aqueous One solution (Promega, Madison, WI) was added to each well, and the cells were incubated for an additional 2 h at 37°C, 7% CO_2_. CellTiter monitors metabolically active cells (indicated by a color change), a property that is lost with dead cells. The colorimetric reaction was measured at 492 nm. In other experiments, treated cells were exposed to 1 ug/ml of propidium iodide (PI) (Sigma Aldrich, St. Louis, MO) for 30 min at 37°C, 7% CO_2_. Cellular damage was evaluated based on the level of PI absorbance (excitation at 536 nm and emission at 617 nm).

## Results

### Development of Immortal Human Pre-adipocytes

**Figure 1:**
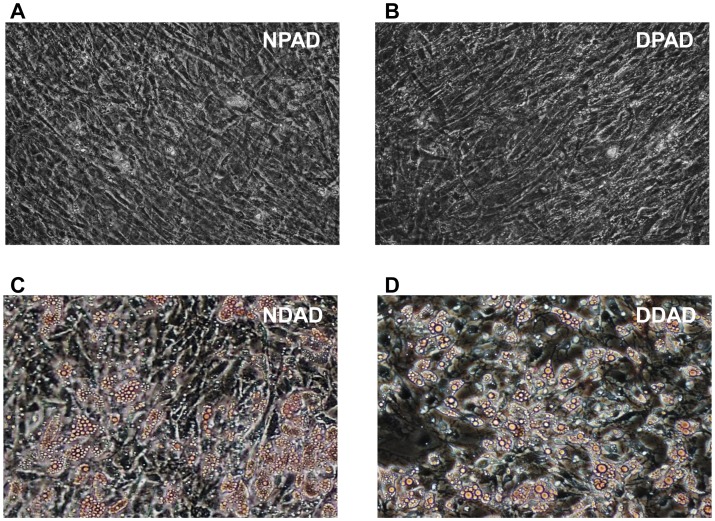
Immortalized preadipocytes accumulate triglyceride droplets upon differentiation. Immortalized non-diabetic preadipocytes (NPADs) and diabetic preadipocytes (DPADs) were incubated with either growth media or differentiation media for 10 days. AdipoRed was used to stain intracellular fat droplets. Undifferentiated NPADs (a) and DPADs (b) did not show triglyceride droplet accumulation, while the differentiated NDADs (c) and DDADs (d) accumulated triglyceride droplets intracellularly. Photographs were taken using a 20X objective (200X magnification).

Primary human pre-adipocytes senesce within a few passages after they are established in culture, which makes it difficult to obtain consistent and reproducible results when assessing the effects of bacterial agents on their inflammatory responses. To overcome this obstacle, we immortalized primary human subcutaneous preadipocytes obtained from both a diabetic and a non-diabetic donor. Immediately after purchase and thawing, the primary pre-adipocytes were co-transduced with retroviruses expressing E6 and E7 proteins of human papillomavirus 16 (HPV 16) along with the reverse transcriptase component of human telomerase (hTERT). After selection, the surviving cells remained culturable without any significant modifications in cell proliferation characteristics until at least passage 30 (∼60 population doublings). For purposes of this study, both of these cell lines are considered immortal and were named DPADs (diabetic pre-adipocytes) and NPADs (non-diabetic pre-adipocytes) (Figure 1a, b).

### Immortal Pre-adipocytes Retain Ability to Differentiate into Adipocytes

Studies were performed to ensure that the immortalized DPADs and NPADs retained properties and characteristics of non-immortalized preadipocytes. The DPAD and NPAD cells were differentiated as described in the Materials and Methods by growing to confluency and adding differentiation agents (i.e. IBMX, indomethacin, dexamethasone, and extra insulin; Lonza). The differentiated cells will be referred to as DDADs (diabetic differentiated adipocytes) and NDADs (non-diabetic differentiated adipocytes). After 10 days of differentiation, DDAD and NDAD cells were stained with AdipoRed dye, which incorporates as a red color in visible triglyceride droplets (Figure 1c, d). Most of the differentiated cells contained large numbers of triglyceride droplets similar to those observed in observed in primary adipocytes [27].

**Figure 2:**
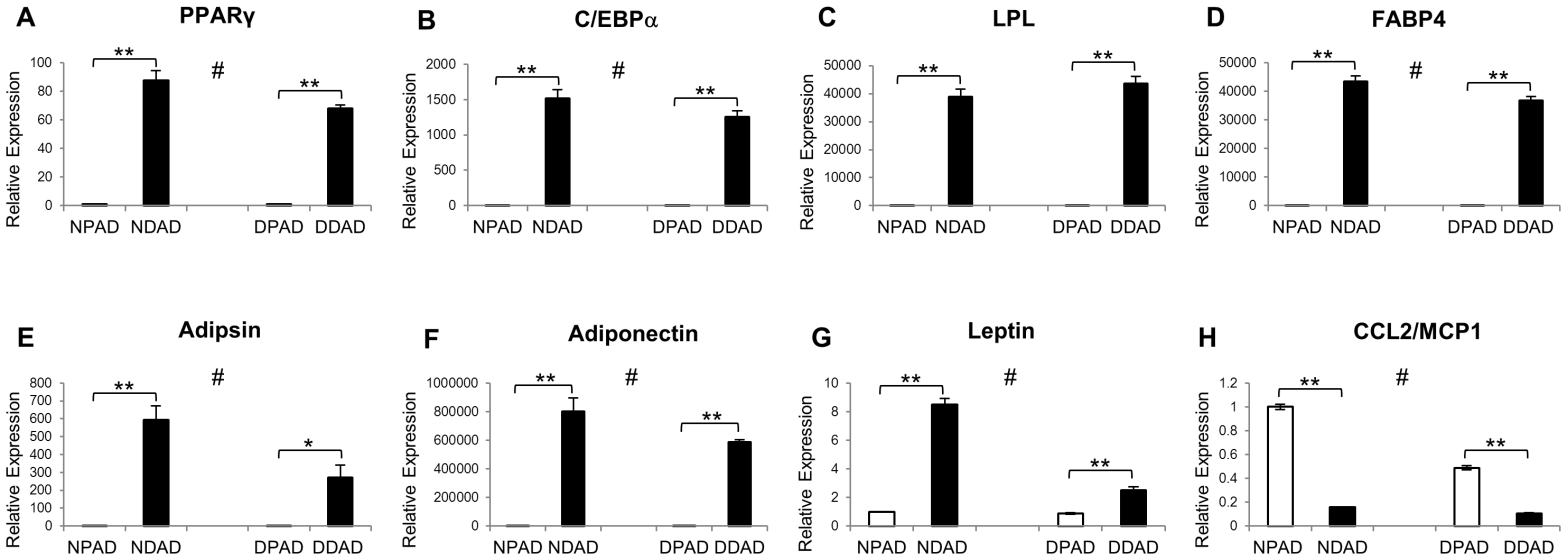
Expression of adipocyte markers in differentiated cells. NPADs and DPADs (white bars) were differentiated into NDADs and DDADs (black bars), respectively. The expression of adipocyte markers was examined by quantitative reverse transcriptase PCR (q-RT-PCR). (A) Peroxisome proliferator-activated receptor gamma (PPARγ); (B) CCAAT/enhancer binding protein-alpha (C/EBPα); (C) lipoprotein lipase (LPL); (D) fatty acid binding protein 4 (FABP-4); (E) adipsin; (F) adiponectin; and (G) leptin were all induced to high levels upon differentiation. (H) A marker of pre-adipocytes termed C-C motif chemokine 2 (CCL2) was expressed in the undifferentiated DPAD and NPAD cells but reduced upon differentiation. Values were normalized to GAPDH and made relative to undifferentiated NPADs and represent 3 replicates. All transcript level changes in differentiated cells as compared to undifferentiated cells were statistically significant (*represents p<0.05; **represents p<0.01, Students t-test). For all genes except leptin, differences between DDADs and NDADs were significant (#p<0.05, Students t-test).

To further assess differentiation of the cells, the expression of adipocyte markers was examined by quantitative reverse transcriptase PCR (q-RT-PCR). The adipocyte markers peroxisome proliferator-activated receptor (PPARγ), CCAAT/enhancer binding protein-α (C/EBPα), lipoprotein lipase (LPL), and fatty acid binding protein 4 (FABP-4) [21], [27] were significantly induced and at similar but not always identical levels in the DDAD and NDAD cultures compared to the undifferentiated paired cells (Figure 2a, b, c, and d). Other markers of adipocyte differentiation including adipsin, adiponectin, and leptin were also induced to high levels upon differentiation with less induction in the differentiated DDAD cells as compared to the NDAD cultures (Figure 2e, f, and g). Some of these markers (e.g. LPL, FABP4, and Adiponectin) were at barely detectable levels in the pre-adipocyte condition but induced 30,000–80,000 fold upon differentiation. On the other hand, a marker of pre-adipocytes termed C-C motif chemokine 2 (CCL2) that is repressed by PPARγ [28] was expressed in the undifferentiated DPAD and NPAD cells but significantly reduced upon differentiation, as expected, in both DDAD and NDAD cultures (Figure 2h).

Collectively, these results demonstrate that both DPADs and NPADs were able to differentiate into adipocytes, with robust expression of primary adipocyte markers and acquisition of adipocyte morphology as previously published [29].

### Inflammatory Response of Adipocytes to Endotoxin

**Figure 3:**
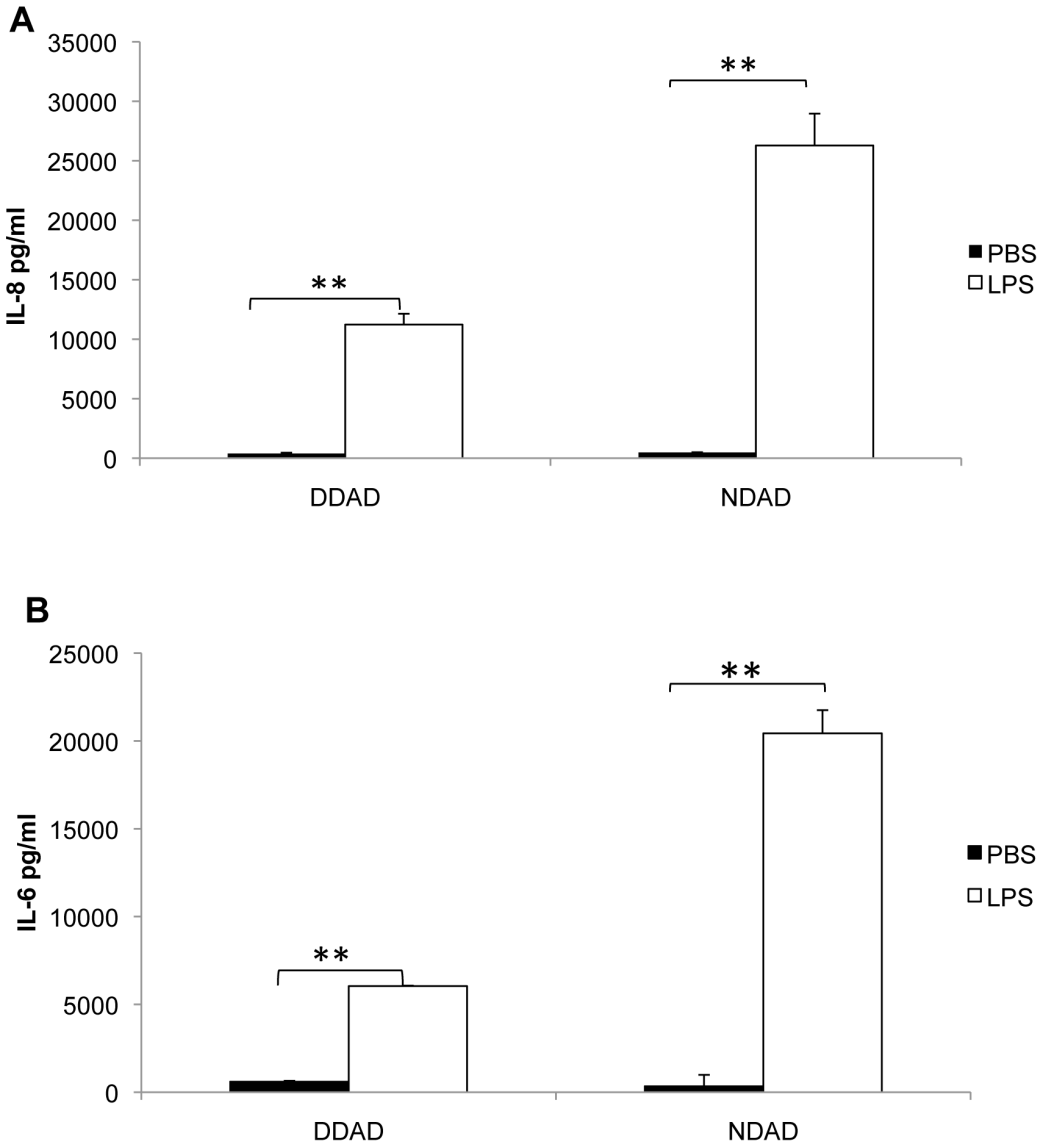
Endotoxin (LPS) induced IL-6 and Il-8 production in both DDADs and NDADs. After differentiation, DDADs and NDADs were treated with endotoxin (5 ng/ml) for 24 h. The culture supernates were collected for IL-6 and IL-8 quantification by ELISA. For both IL-6 and IL-8, relative differences between DDADs and NDADS were significant (**represents p<0.01, Students t-test).

Since previous studies showed that primary human subcutaneous adipocytes respond to endotoxin (LPS) by up-regulating production of IL-6 and IL-8, the effects of endotoxin on DDADs and NDADs were examined. Differentiated cells were exposed to 5 ng/ml of endotoxin for 24 h. The culture supernates were then collected for measurement of IL-6 and IL-8, as representative pro-inflammatory cytokines. As expected, endotoxin significantly induced IL-6 and IL-8 responses in both DDAD and NDAD cells, with higher relative induction in NDADs (Figure 3). Our data indicate that endotoxin induces inflammatory cytokine and chemokine production in DDAD and NDAD cells to a similar degree as in primary cells (5).

### Effects of SAgs on Adipocytes

In previous studies, we have shown that *S. aureus* SAgs have major roles in multiple diseases, such as TSS, necrotizing pneumonia, and infective endocarditis [9]. In addition, SAgs, like TSST-1, induce pro-inflammatory responses in human vaginal epithelial cells [13]. It is established in obese and diabetic patients that adipocytes induce the innate immune system via production of pro-inflammatory signals, contributing to chronic systemic inflammatory states and leading to glucose tolerance and insulin resistance [1], [3]. *S. aureus* colonizes a large percentage of humans on mucosal and skin surfaces, and the organism is the most common isolate in diabetic foot ulcer infections, which is highly associated with obesity (especially in the case of DMII). Since all human strains of *S. aureus* we have tested produce high-levels of SAgs, the pro-inflammatory effects of SAgs on adipocytes were assessed.

**Figure 4:**
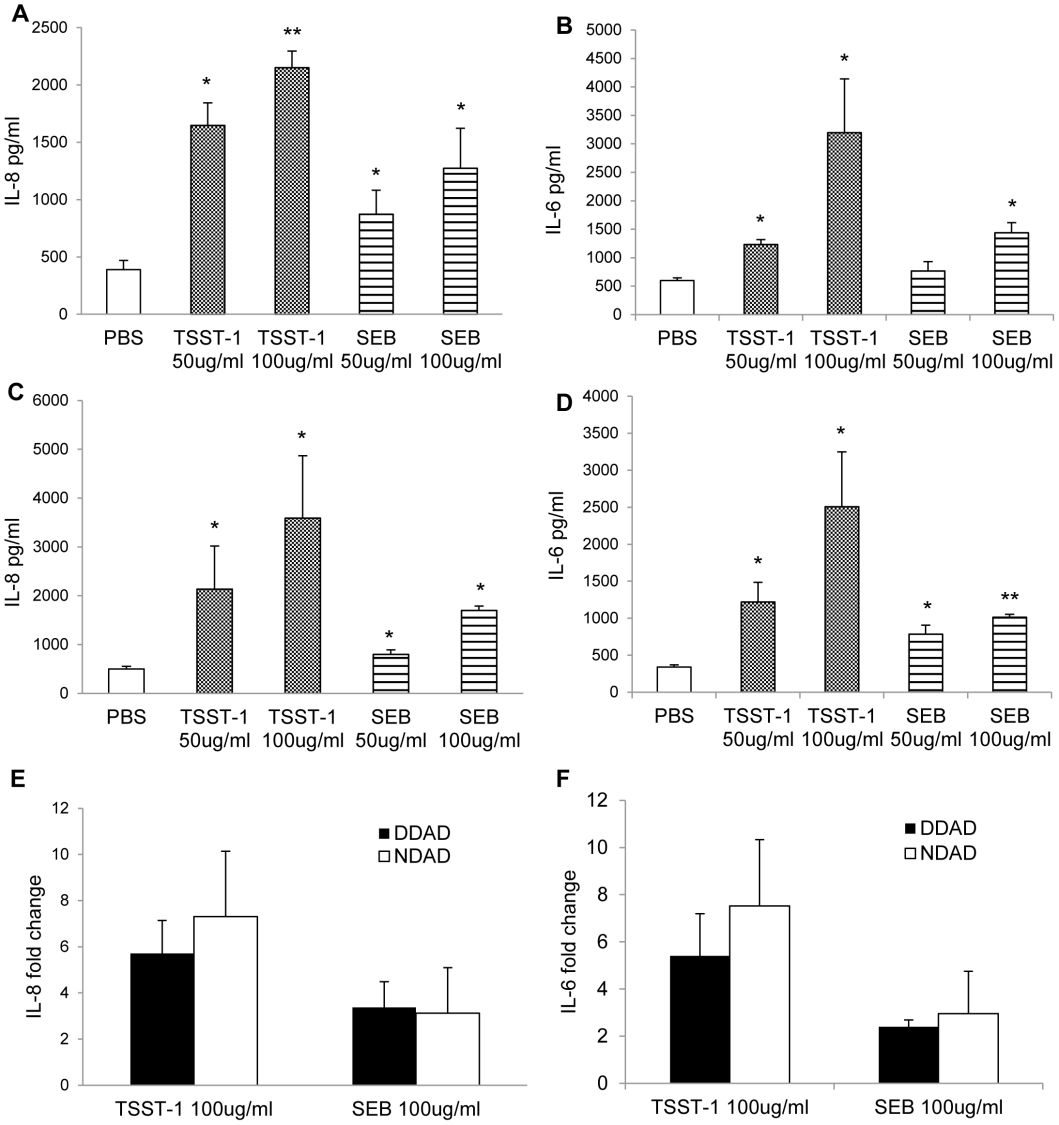
TSST-1 and SEB induced IL-6 and Il-8 production in DDADs and NDADs. Differentiated adipocytes were treated with 50/ml and 100 ug/ml of TSST-1 or SEB for 24 h. The culture supernates were collected from DDADs for IL-6 (a) and IL-8 (b) and NDADs IL-6 (c) and IL-8 (d) for quantification by ELISA (*represents p<0.05; **p<0.01, Students t-test). Fold changes in IL-6 (e) and IL-8 (f) production were calculated in DDADs and NDADs after TSST-1 and SEB exposure. Values represent fold changes relative to PBS-treated controls.

DDAD and NDAD cells were treated with two physiologically-relevant concentrations (50 ug/ml and 100 ug/ml) of the SAgs TSST-1 and staphylococcal enterotoxin B (SEB) for 24 h, a time point that we found in preliminary studies to give the best response. The culture supernates were collected and IL-6 and IL-8 were quantified by ELISA. Both TSST-1 and SEB significantly (p<0.05) induced IL-6 and IL-8 production in DDAD and NDAD cells in a dose dependent manner (Figure 4a, b, c, d). It has also been reported that adipose tissue from normal and diabetic individuals may respond differently to endotoxins [6]. However, we saw no significant difference in relative IL-6 and IL-8 production between NDAD and DDAD cell lines after exposure to TSST-1 or SEB (Figure 4e, f). Collectively, these results indicate that, like endotoxin, SAgs significantly induced proinflammatory response in both adipocyte lines.

### Additive Effects of SAgs and Endotoxin on Adipocytes

**Figure 5:**
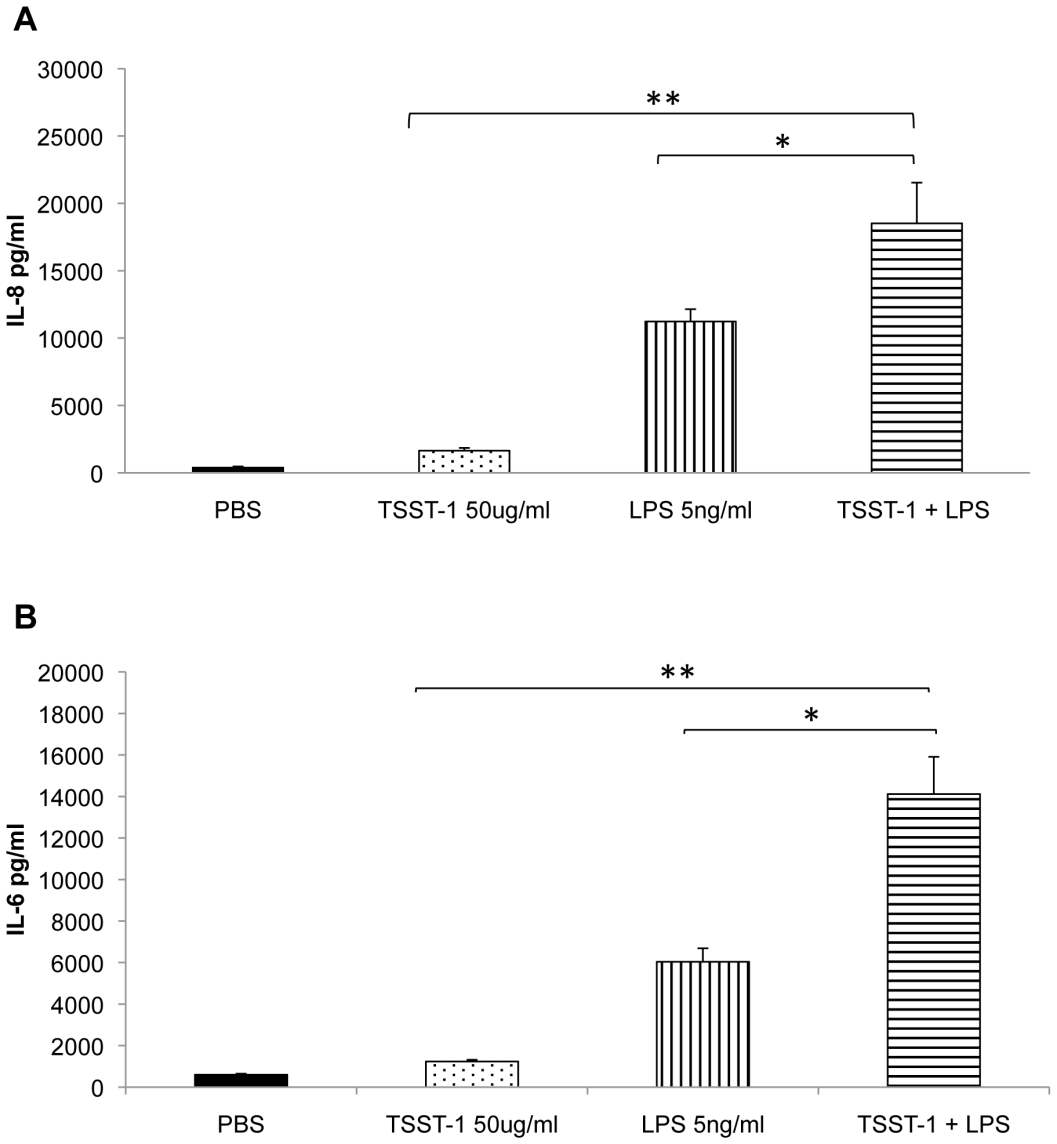
TSST-1 and endotoxin (LPS) cooperatively induce IL-6 and IL-8 production in DDADs. DDADs were treated with TSST-1 (50 ug/ml), endotoxin (5 ng/ml), or TSST-1 (50 ug/ml)+endotoxin (5 ng/ml) for 24 h. The culture supernates were collected for IL-6 and IL-8 quantification by ELISA. TSST-1+ endotoxin induced more IL-6 (a) and IL-8 (b) production in DDADs than PBS, TSST-1, or endotoxin alone (*represents p<0.05; **p<0.01, Students t-test).

SAgs greatly amplify the lethal effects of endotoxin [9], [15], [16]. Thus, the effect of co-administration of both SAgs and endotoxin was examined on DDADs. After 24 h treatment with TSST-1 (50 ug/ml) and endotoxin (5 ng/ml), DDADs upregulated IL-6 and IL-8 production significantly compared to the untreated (PBS) group (Figure 5a, b). Additionally, The IL-6 and IL-8 levels in co-treated DDADs were also significantly higher than those in the groups treated either TSST-1 or endotoxin alone.

### Effects of Staphylococcal α-toxin on Adipocytes

**Figure 6:**
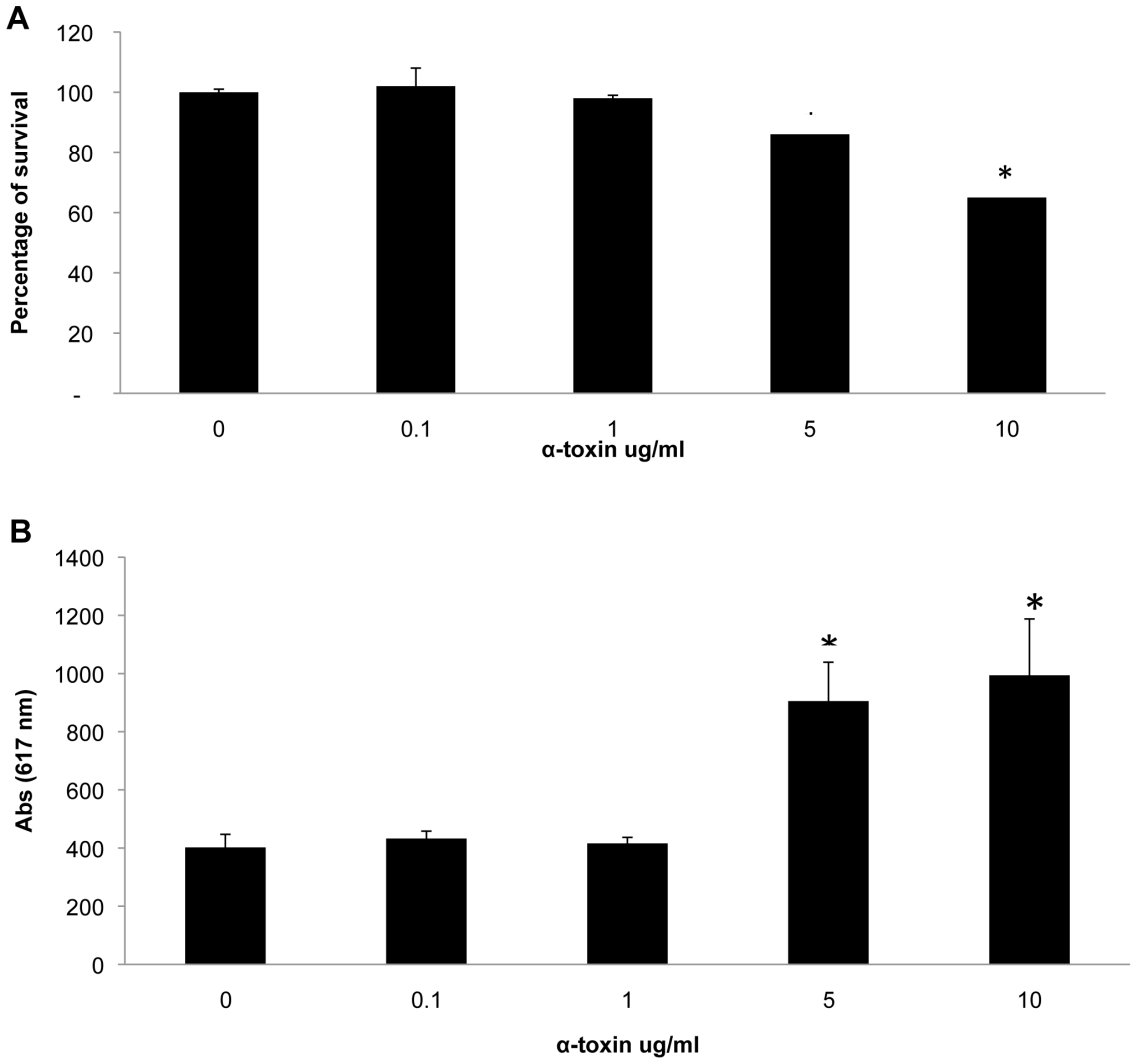
Staphylococcal *α*-toxin was cytotoxic for DDADs. DDAD cells were treated with various concentrations of α-toxin for 24 h. The Cell Titer 96® Aqueous One Solution Proliferation Assay and propidium iodine were used to assess the cellular toxicity and damage. (a) At 5 ug/ml and 10 ug/ml of α-toxin significant cell death occurred, while no effect was observed at lower concentrations (below 1 ug/ml). (b) Consistent with the cytotoxic effect, significant amounts of PI were incorporated into DDADs at 5 ug/ml and 10 ug/ml of α-toxin, but not at lower concentrations (*represents p<0.05, Students t-test).

One of the most pro-inflammatory and cytotoxic molecules of *S. aureus* is the cytolysin α-toxin; the toxin is important in production of skin infections through its dermonecrotic and pro-inflammatory activities [11], [30]. We thus tested the effects of α-toxin on DDADs with cells treated with various concentrations of α-toxin for 24 h at 37°C in 5% CO_2_. Propidium iodide and CellTiter aqueous one solution (Promega) were used to measure cytotoxicity, while the culture supernates were collected for IL-6 and Il-8 measurement by ELISA. At concentrations of 5 ug/ml and 10 ug/ml, α-toxin was significantly cytotoxic, while cytotoxicity was insignificant at lower concentrations (Figure 6).

**Figure 7:**
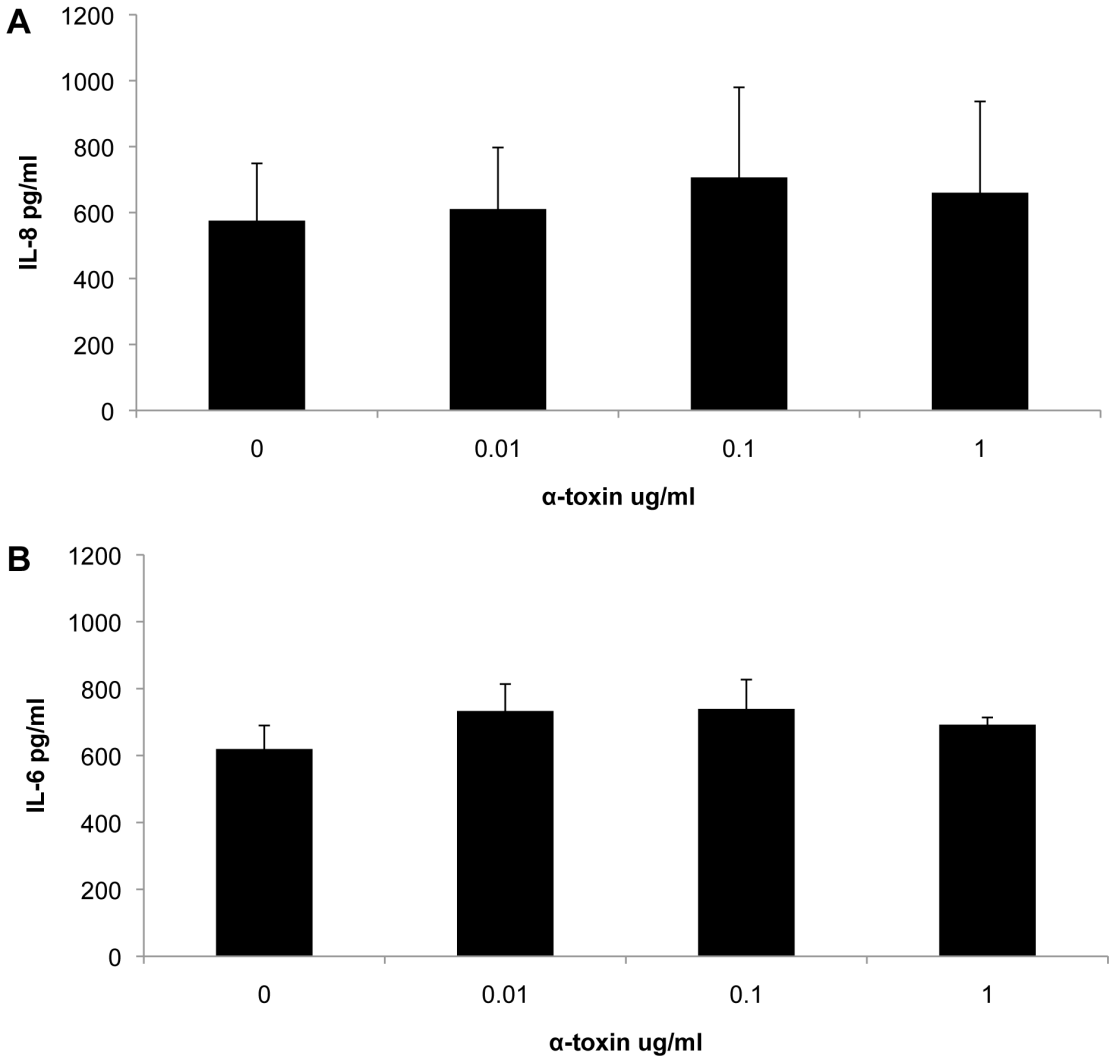
α-toxin failed to induce IL-6 and IL-8 production in DDADs. DDADs were treated with low concentrations of α-toxin for 24 h. The culture supernates were collected for IL-6 (a) and IL-8 (b) quantification by ELISA.

Since α-toxin killed the cells at high concentrations, only culture supernates which were collected from cells treated with low α-toxin concentrations (below 1 ug/ml) were used to measure the amount of released IL-6 and Il-8. There was no difference in the amount of released IL-6 and IL-8 between the control and the α-toxin treated cells (Figure 7).

## Discussion

For this study, we developed diabetic and non-diabetic pre-adipocyte cell lines, DPAD and NPAD, which can be differentiated into adipocytes to assess responses to foreign stimuli such as endotoxin and exotoxins. These cells appear to function normally in that they exhibit contact inhibition when cultured in vitro and can be differentiated into adipocytes that, as expected, accumulate fat drops and properly express adipocyte-specific differentiation genes. The DPAD and NPAD lines provide a valuable resource for studying how bacterial infections and their toxins affect adipocyte inflammatory response and biology. They should also be useful for other purposes such as understanding to role of adipocytes in the development of metabolic syndrome and its associated diseases. Several mouse pre-adipocyte-like cell lines (e.g. 3T3-L1 and 3T3-F442A) have been generated that can readily differentiate into adipocytes (Reviewed in [31], [32]). These rodent cell lines have been valuable for understanding what factors regulate adipocyte differentiation. With regard to bacterial infections and rodent lines, one recent study reported that *S. aureus* can infect mouse 3T3-L1 cells and stimulate MCP-1 and IL-6 production [33], although there was no attempt to determine what *S. aureus* factors were involved in this response. Since mouse and human cells may respond very differently to pathogens such as *S. aureus*, it is important to use human cells. Primary human pre-adipocytes isolated from fat tissue are one possible option for these types of studies, but, unfortunately, primary pre-adipocytes proliferate for only 2 or 3 passages [34]. Human mesenchymal stem cells (MSCs) isolated from a variety of tissues are also capable of being differentiated into adipocytes but, like pre-adipocytes, MSCs have a limited lifespan [35]. In addition, a source for MSCs is often not readily available for most researchers, and initial cultures are a mixed population of cells, which could influence experimental results. In a single published case, normal human pre-adipocytes were immortalized by TERT and HPV-16 E7 [27]. The derived cells, referred to as Chub-S7 cells, were shown to respond to adipocyte-differentiation factors and differentiate into mature adipocytes [36]. For unknown reasons, reports on the use of Chub-S7 are limited in the literature, and they have never been used to assess the effects of bacterial infection or factors on adipocyte inflammatory response or function. In our studies, the DPAD and NPAD cells that we generated were immortalized by a combination of TERT and HPV-16 E6 and E7. This combination of genes allows immortalization without crisis, which may be important for preserving differentiation capacity. Indeed, by using these same factors we have immortalized vaginal epithelial cells and airway epithelial cells that retain differentiation capacity and normal function for many passages [13], [37]. In the present studies, we found that the DPAD and NPAD cells retain their ability to differentiate into mature adipocytes for at least 30 population doublings but, as with any continuous cell line, it is possible that they will lose this capacity over time and studies are underway to address this possibility.

By using the DPAD and NPAD cells that we developed, we have shown that adipocytes from diabetic and non-diabetic individuals respond to foreign antigens (endotoxin and SAgs) to produce pro-inflammatory cytokines such as IL-6 and IL-8. It has previously been shown that endotoxin exhibits these cytokine-producing properties with stimulation of adipocytes [4], [5], [38]. Chronic exposure to endotoxin may lead to insulin resistance and promote development of DMII [4], [38]. We have now extended these findings to include staphylococcal SAg stimulation of adipocytes to produce the same pro-inflammatory cytokines. Additionally, we have shown that SAgs synergize with endotoxin to induce production of cytokines.

A previous study has suggested that a more robust inflammatory response to endotoxin occurs in subcutaneous fat tissue from type II diabetic as compared to non-diabetic donors [6]. However, we did not observe this differential effect in our studies using diabetic and non-diabetic adipocyte cell lines. Treatment of the differentiated cells with TSST-1 or SEB yielded a similar inflammatory response and, if anything, the response to endotoxin was dampened in the diabetic cells. There are several potential explanations for these differences. For one, we used cell lines that were purely adipocytes whereas the previous studies used adipocyte tissue, which would include other inflammatory cells types that may influence the response. In addition, there could be significant variability between different tissue donors and derived cell lines. Any claim of a difference in inflammatory responses between diabetic and non-diabetic adipocytes would have to be validated using multiple tissues and/or cell lines from different donors.

Staphylococcal SAgs and α-toxin are highly pro-inflammatory to human vaginal epithelial cells [23]. As noted above, SAgs are also stimulatory to adipocytes. Surprisingly, α-toxin is not. In prior studies, we have shown that nearly all pathogenic *S. aureus* strains produce high levels of SAgs [9], [39]. Thus, persons who are colonized with *S. aureus* are being exposed to SAgs. At any given time, as many as 40% of persons have *S. aureus* nasally; however, nearly all persons are exposed to *S. aureus* [39], [40], [41], [42]. Nearly all persons with damaged skin, whether atopic dermatitis or diabetic foot ulcers, become colonized with *S. aureus* and are thus exposed to SAgs [39], [42]. Additionally, it has been established that diabetic patients have increased infections rates overall to a variety of microbes including *S. aureus* [43], [44], [45], [46]. Staphylococcal SAgs amplify the lethal effects of gram-negative endotoxin by up to 10^6^-fold [40] and synergize with SAgs in cytokine production systemically as tested in experimental animals [16]. Thus, low-level daily exposure of persons to endotoxin through leakage across the intestinal tract would be expected to have greater effects on humans through synergy with SAgs. Such an effect could accelerate the processes leading to insulin resistance and poor wound healing responses in DMII persons.

The mechanism by which *S. aureus* toxins stimulate proinflammatory cytokine production in adipocytes is, at this time, unknown. Previous studies have shown that adipocytes express toll-like receptor (TLR)4 and TLR2 and that adipocytes respond to LPS to induce cytokine secretion [5], [47], [48], [49]. It has been speculated that activation of TLRs in adipocytes may be one mechanism by which an inflammatory response is induced and insulin resistance is initiated and sustained in the development of metabolic syndrome [28], [48], [50]. *S. aureus* SAgs may activate the TLR receptors in adipocytes or activation of TLRs by LPS may cause upregulation of SAg receptors, which could potentially explain the synergy between LPS and SAgs that we observed. Further experiments will be necessary to address this possibility. TSST-1 and SEB SAgs are known to bind to MHC class II molecules, HLA-DR and DQ [51], [52]. Adipocytes have been shown to express MHC class II molecules and to induce the migration of CD4+ T cells when stimulated by LPS [53]. Whether MHC class II molecules play a role in induction of an inflammatory response by SAgs in adipocytes is unknown. In a previous report, we have also speculated that TSST-1 might bind to and activate CD40 [54]. Interestingly, it has been demonstrated that mature adipocytes express CD40 that can be activated, by CD40 ligand or activated CD4+ T cells, to induce secretion of inflammatory cytokines [55]. Further studies will be necessary to specifically identify what receptors on adipocytes are responding to SAgs and how this leads to activation of an inflammatory response.

In summary, we have developed two immortalized preadipocyte cell lines that were used to study cytokine production as induced by exposure to endotoxin, SAgs, and endotoxin+SAgs; all combinations caused cytokine production with synergy seen between endotoxin and SAgs. Our studies suggest another mechanism whereby the microflora may be involved in the development of metabolic syndrome and its associated clinical problems.
